# RACIAL DISPARITIES IN HOSPICE USE AT THE END OF LIFE: FINDINGS FROM THE REGARDS STUDY

**DOI:** 10.1093/geroni/igz038.1632

**Published:** 2019-11-08

**Authors:** Katherine Ornstein, Orla C Sheehan, Jin Huang, J David Rhodes, David L Roth

**Affiliations:** 1 Icahn School of Medicine at Mount Sinai, New York, New York, United States; 2 Center on Aging and Health, Johns Hopkins University School of Medicine, Baltimore, Maryland, United States; 3 Johns Hopkins University School of Medicine, Baltimore, Maryland, United States; 4 School of Public Health, Department of Biostatistics, Birmingham, Alabama, United States

## Abstract

Hospice supports patients and families through interdisciplinary care focused on symptom management and maximization of quality of life. Although hospice care confers well-documented benefits, it remains underutilized: many patients do not use it at all or enter care too late to receive any benefit. While racial disparities in hospice use have been documented, hospice utilization among non-white decedents remains understudied, particularly among those with non-cancer diagnoses. Therefore, we used the REasons for Geographic and Racial Differences in Stroke (REGARDS) study, a population-based investigation of stroke incidence with oversampling of Blacks and cause of death adjudication by expert panel review, linked to Medicare claims data to examine racial disparities in end-of-life care. We identified 1221 participants who died between 2013-2015 due to natural causes excluding sudden death. More than half (52.8%) used hospice during the last 6 months of life (median =15 days), with use among cancer decedents over 70%. Overall, Blacks were significantly less likely to use hospice (OR=0.570) compared to Whites in adjusted analyses. Among hospice users, Blacks did not significantly differ from Whites in length of stay. In analyses stratified by cause of death (dementia, cancer, CVD and other), Blacks were significantly less likely than Whites to use hospice for all causes of death other than dementia. Despite tremendous growth of hospice in recent decades, our findings suggest that this effective service remains highly underutilized among Blacks dying from cancer, CVD and other serious illnesses, suggesting a need for targeted intervention to eliminate disparities in quality end-of-life care.

